# Reproductive effects of sulfoxaflor in male Sprague Dawley rats

**DOI:** 10.1007/s11356-022-19006-3

**Published:** 2022-02-11

**Authors:** Wafaa H. Mohamed, Marwa F. Ali, Doha Yahia, Hassan A. Hussein

**Affiliations:** 1 Department of Forensic Medicine and Toxicology, Faculty of Veterinary Medicine, Aswan University, Aswan, Egypt; 2 Department of Veterinary Pathology and Clinical Pathology, Faculty of Veterinary Medicine, Assiut University, Assiut, Egypt; 3 Department of Forensic Medicine and Toxicology, Faculty of Veterinary Medicine, Assiut University, Assiut, Egypt; 4 Department of Theriogenology, Faculty of Veterinary Medicine, Assiut University, Assiut, Egypt

**Keywords:** Sulfoxaflor, Reproductive toxicity, Sperm viability, Sperm morphology and sperm DNA damage

## Abstract

The study objective was to evaluate the potential reproductive toxicity of sulfoxaflor (SFX) insecticide in male Sprague Dawley rats. To attain these objectives, forty male Sprague Dawley rats of 10–12 weeks old were randomly divided into four equal groups; the 1st group was used as a control group; the other three groups were exposed to 25, 100, and 500 mg/kg body weight SFX by oral gavage for 4 weeks. Relative testicular weight, testosterone, FSH, LH, MDA, and GPx levels, sperm viability, sperm morphology, sperm DNA damage, and histopathological changes in testes, epididymis, and seminal vesical of these rats were investigated after 4 weeks. The results showed that SFX exposure resulted in a significant increase in FSH, LH, MDA, and GPx levels as well as the percentage of dead and abnormal sperms and DNA damage in rat sperms. Histopathological examination of testes established testicular degeneration with coagulative necrosis as well as the proliferation of interstitial connective tissue infiltrated with inflammatory cells with congestion of intertubular blood vessels in epididymis and degeneration of lining epithelium of seminal vesicles.

## Introduction

The use of pesticides in global agriculture is uncontrolled and poses a huge threat to public health. Many investigations have revealed that the presence of the residues of pesticides in soil, water, and agricultural products can affect humans and can enter to the bloodstream and bind to circulating serum proteins **(**Hadi Chegni et al. 2020**)**. Insect resistance to the existing insecticides is a big problem and has cost billions of dollars in the agricultural field and could result in many insect-vectored diseases **(**Gould et al. 2018**)**. Although many species of insect are controlled by neonicotinoids, but their extensive use has led to the presence of resistance in some pests as sap-feeding insects **(**Bass and Field 2018**)**. Sulfoximine is a relatively recent insecticidal designed to control of sap-feeding insects **(**Watson et al. 2021**)**. Sulfoxaflor (SFX) is the first compound in this category and was selected for commercial development. Sulfoximine (SFX) acts on nicotinic acetylcholine receptors (nAChRs) in insects and it functions in a different mode from other insecticides acting at nAChRs **(**Sparks et al. 2020**)**. It belongs to the fourth generation of neonicotinoid; it has been regarded as subgroup 4C by the Insecticide Resistance Action Committee (IRAC) due to its sulfoximine structure and low insecticidal resistance (Xu et al. 2020).

Its residues would stay in the plants, water, and soil for many days after treatment; therefore, the toxicity of SFX may increase due to its long-term bioaccumulation through the food chain (Xu et al. 2020). In spite of the fact that SFX has more advantages than other insecticides, it can alter nicotinic acetylcholine receptors, causing neurotoxicity (Authority et al. 2019) and induced liver effects as hepatocellular hypertrophy and liver tumors in rats and mice **(**Bacci et al. 2018**)**, although several studies reported the toxic effects of different neonicotinoids on animals and humans, such as hepatotoxicity, neurotoxicity, and immunotoxicity **(**Fathy and Abdelkader 2021a; Piner Benli and Çelik 2021b**)**. However, the reproductive effects of SFX have not been evaluated; yet, therefore, the goal of the current study is to investigate the impact of sulfoxaflor on the reproductive system of Sprague Dawley rats.

## Materials and methods

### Chemicals

A commercial formulation of SFX (Dow AgroSciences Company, Canada) as a suspension containing 24% (w/w) sulfoxaflor as an active ingredient, sodium carbonate (El Nasr Pharmaceutical Chemicals Co., Egypt), methyl violet, eosin, and nigrosin stain (S. D Fine-Chem Limited, India). All chemicals and reagents were of the highest level of purity.

### Animals

A total of 40 male Sprague Dawley rats (10–12 weeks old weighing 120–150 g) were purchased from the Animal House, Faculty of Medicine, Assiut University. Rats were kept in plastic cages that contained five rats each. Animals were kept for 2 weeks before the experiment for acclimatization. Feed and drinking water were administered ad libitum. Rats were maintained at a temperature of 22 ± 2 °C, and a lighting period of 12 h (light/dark). The handling of laboratory animals was carried out in accordance with the international guidelines of the National Research Council (NRC 1996). Protocols of the study were approved by the ethical committee of the Faculty of Veterinary Medicine, Assiut University, Egypt (March, 2021).

### Experimental design

Forty rats were divided into four groups of 10 animals each:

Group I Control group**,** rats were given distilled water 1 ml/100 g orally using stomach tube day by day for 4 weeks.
Group II Rats were exposed to SFX (dissolved in distilled water) at a dose rate 25 mg/kg body weight by oral gavage, day by day for 4 weeks. This dose is 1/40 of the oral LD_50_ of SFX (1000 mg/kg body weight) as reported by FAO/WHO (2003).
Group III Rats were administered SFX at a dose rate 100 mg/kg body weight which was 1/10 LD_50_ by oral gavage, day by day for 4 weeks.
Group IV Rats were dosed SFX at a dose rate 500 mg/kg body weight which was 1/2 LD_50_ by oral gavage, day by day for 4 weeks.

### Samples collection

At the end of the experiment, ten rats from each group were euthanized by diethyl ether anesthesia for samples collection. Blood samples were collected from the descending aorta into sterile test tubes without anticoagulants. Serum was obtained after centrifugation at 3000 rpm for 10 min. The serum samples were kept at − 20 °C to estimate testosterone, FSH, and LH hormones levels and evaluate oxidative status by measuring malondialdehyde (MDA) and glutathione peroxidase (GPx). The caudal part of the epididymis was cut and transferred into petri dish containing drops of normal saline at 37 °C then a few drops of sperm suspension were taken to evaluate the viability, morphology, and DNA integrity of sperms. For histopathological examination, samples from testes, epididymis, and seminal vesical were fixed in 10% formaldehyde, dehydrated, and embedded in paraffin then sections were cut and stained with hematoxylin and eosin (H&E) (Suvarna et al. 2018).

### Organosomatic index of testes

The total body weight and testicular weight were determined and the testicular organosomatic index was calculated (testicular weight/ body weight).

### Hormonal assays

#### Serum testosterone levels

The concentration of serum testosterone was estimated at wavelength 450 nm according to the method of Tietz (1995) using a testosterone ELISA test kit (Biodiagnostic, Egypt) by ELISA reader apparatus (Bio-Tec instrument).

#### Serum follicle stimulating hormone levels

Levels of serum FSH were determined according to Scott et al. (1989) at 490–630 nm using an enzyme immunoassay test kit which was obtained from Biodiagnostic, Egypt. ELISA reader apparatus used is Bio-Tec instrument.

#### Serum luteinizing hormone levels

Serum LH levels were estimated according to Beastall et al. (1987) at 490–630 nm using an enzyme immunoassay test kit which was purchased from Biodiagnostic, Egypt. ELISA reader apparatus used is Bio-Tec instrument.

### Estimation of serum MDA level and GPx activity

MDA level was measured at 534 nm by the method as described by ohkawa et al. (1979). However, GPx activity was estimated at 340 nm according to the method of Paglia and Valentine (1967) using commercial kits (Biodiagnostic, Egypt) and through a spectrophotometer (UV-2100 spectrophotometer, Unico, USA).

### Evaluation of epididymal sperm parameters

#### Sperm viability test

To estimate sperm viability percentage, the cauda epididymal duct was exposed and incised; the semen that oozed was diluted 200 (0.05 μL of sperm with 99.95 μL of PBS) times in physiological saline. In a sterile test tube, 20 μL of sperm suspension was put with 20 μL of 0.05% eosin-Y. After 20 s, 50 μL of nigrosin was mixed thoroughly with them. The mixture of stained sperm was smeared on the slide and examined under a light microscope (Olympus BX 43, Japan) at × 100. For each slide, a total of 200 sperm cells were examined. Dead sperms were pink and live sperms were not stained. This method was described by Wyrobek et al. (1983).

#### Sperm morphology test

The percentage of morphologically abnormal spermatozoa was determined according to the method of Menkveld (2010). The sample was diluted with 1% sodium chloride solution. After thoroughly mixing, one drop of sperm suspension was smeared on a slide. Dried in the air and stained immediately with one part of a 1% sodium carbonate solution is mixed thoroughly with nine parts of 1% the methyl-violet solution immediately before use. The stain was left for 4 to 5 min. The preparation was then washed with distilled water. The slide dried with filter paper and sperm cells were examined under a light microscope at × 100 magnification.

#### Assessment of sperm DNA damage

A small drop of the sperm suspension was smeared on the glass slides. The slides were dried in air and fixed in Carnoy’s solution (methanol/acetic acid, 3:1) for 2 h. The slides were stained for 5 min with freshly prepared acridine orange solution (19.00%) in phosphate citrate. The slides were washed with deionized water, then examined using a fluorescent microscope (Olympus BX43, Japan) equipped with a blue filter. Two hundred sperms were examined on each slide. Sperm heads with intact chromatin had green fluorescence, while those with denatured chromatin had orange-red staining (Tejada et al. 1984).

### Histopathological investigation

Fresh specimens from testes, epididymides, and seminal vesicles of rats of all experimental groups were collected and fixed in 10% neutral buffered formalin. Tissue samples were processed routinely, sectioned at 4 µm thickness, and stained with hematoxylin and eosin (H&E) for histopathological examination by light microscopy (Olympus, CX31; Tokyo Japan) and photographed using a digital camera (Toupview, LCMos10000KPA, China) (Suvarna et al. 2018). The microscopic findings for each group were presented in a table to demonstrate the type of lesion, severity, and percentage of animals according to Sayed et al. (2014).

### Data analysis

The results were presented as mean ± SE. One-way analysis of variance (ANOVA) was used followed by post hoc test (Tukey) to detect a significant difference among groups. These analyses were conducted using the SPSS program for Windows, version 16.0 (Borenstein et al. 1997). Differences between and among the groups were considered significant at *p* ≤ 0.05.

## Results

No death occurred in any experimental group throughout the experiment.

### Testicular somatic index

The relative testicular weight did not show significant changes in the exposed groups in comparison with the control group (Table 1).

**Table 1:** Testicular organosomatic index in rats after oral administration of SFX (25, 100 and 500 mg/kg/b.w.) for 4 weeks

Groups	Testicular organosomatic index
Control	2.140 ± 0.133
25 mg/kg SFX	1.875 ± 0.094
100 mg/kg SFX	1.655 ± 0.194
500 mg/kg SFX	1.382 ± 0.330

### Hormonal assays (serum testosterone, FSH, and LH levels)

Testosterone level in serum did not illustrate any significant change at any dose when compared to the control group. After 4 weeks of treatment, SFX at 500 mg/kg caused a significant (*P* < 0.01) increase in FSH level in comparison with the control and 25 mg/kg dose groups, while LH level significantly increased in SFX-exposed groups to 100 mg/kg (*P* < 0.05) and 500 mg/kg (*P* < 0.01) as compared with the control group and SFX at 500 mg/kg showed a significant (*P* < 0.01) increase as compared with 25 mg/kg group. Also, there was a significant (*P* < 0.01) increase in LH level in rats exposed to 500 mg/kg in comparison with rats exposed to 100 mg/kg (Table 2).

**Table 2:** Testosterone, FSH, and LH hormones levels in rats after oral administration of SFX (25, 100, and 500 mg/kg/b.w.) for 4 weeks

Groups	Testosterone (ng/mL)	FSH (µl U/mL)	LH (µl U/mL)
Control	3.150 ± 0.144	0.250 ± 0.005	1.510 ± 0.017
25 mg/kg SFX	3.266 ± 0.290	0.270 ± 0.015	1.636 ± 0.080
100 mg/kg SFX	3.400 ± 0.173	0.326 ± 0.035	1.776 ± 0.054ac*
500 mg/kg SFX	3.686 ± 0.121	0.426 ± 0.027a*b*	2.110 ± 0.017a*b*c*

Values were expressed as means ± SE (*n* = 10). * indicates highly significant at *p* ≤ 0.01; a indicates significance at *p* ≤ 0.05 in comparison with the control group; b indicates significance at *p* ≤ 0.05 in comparison with 25 mg/kg dose group; c indicates significance at *p* ≤ 0.05 between 100 and 500 mg/kg dose groups

### MDA level and GPx activity

The level of MDA significantly (*P* < 0.01) increased in all SFX-exposed groups as compared with the control group, while 500 mg/kg group revealed a significant (*P* < 0.01) increase in comparison with 25 and 100 mg/kg dose groups. GPx activity illustrated a significant (*P* < 0.01) increase in 100 and 500 mg/kg dose groups as compared with the control group and 25 mg/kg dose group. However, GPx in rats exposed to 500 mg/kg SFX showed a significant (*P* < 0.01) increase when compared with 100 mg/kg dose group (Table 3).

**Table 3:** MDA level and GPx activity in rats exposed to 25, 100, and 500 mg/kg/b.w.of SFX for 4 weeks

Groups	MDA (nanomole/mg protein)	GPx (U/mL)
Control	0.220 ± 0.011	117 ± 1.154
25 mg/kg SFX	0.365 ± 0.008 a*	130 ± 1.154
100 mg/kg SFX	0.396 ± 0.018 a*c*	246 ± 5.773 a*b*c*
500 mg/kg SFX	0.523 ± 0.026 a*b*c*	306 ± 4.618 a*b*c*

Values were expressed as means ± SE (*n* = 10). * indicates highly significant at *p* ≤ 0.01; a indicates significance at *p* ≤ 0.05 in comparison with the control group; b indicates significance at *p* ≤ 0.05 in comparison with 25 mg/kg dose group; c indicates significance at *p* ≤ 0.05 between 100 and 500 mg/kg dose groups

### Sperm parameters

**Fig. 1:**
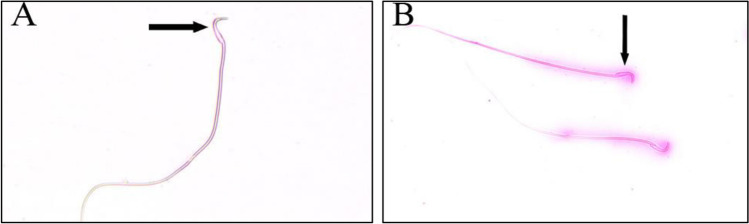
**A** Eosine nigrosin-stained semen film of control rats showed normal live epididymal sperm, not stained (arrow). **B** Semen film of SFX-treated rats showed dead epididymal sperms (appeared pink) (arrow) (× 40)

**Fig. 2:**
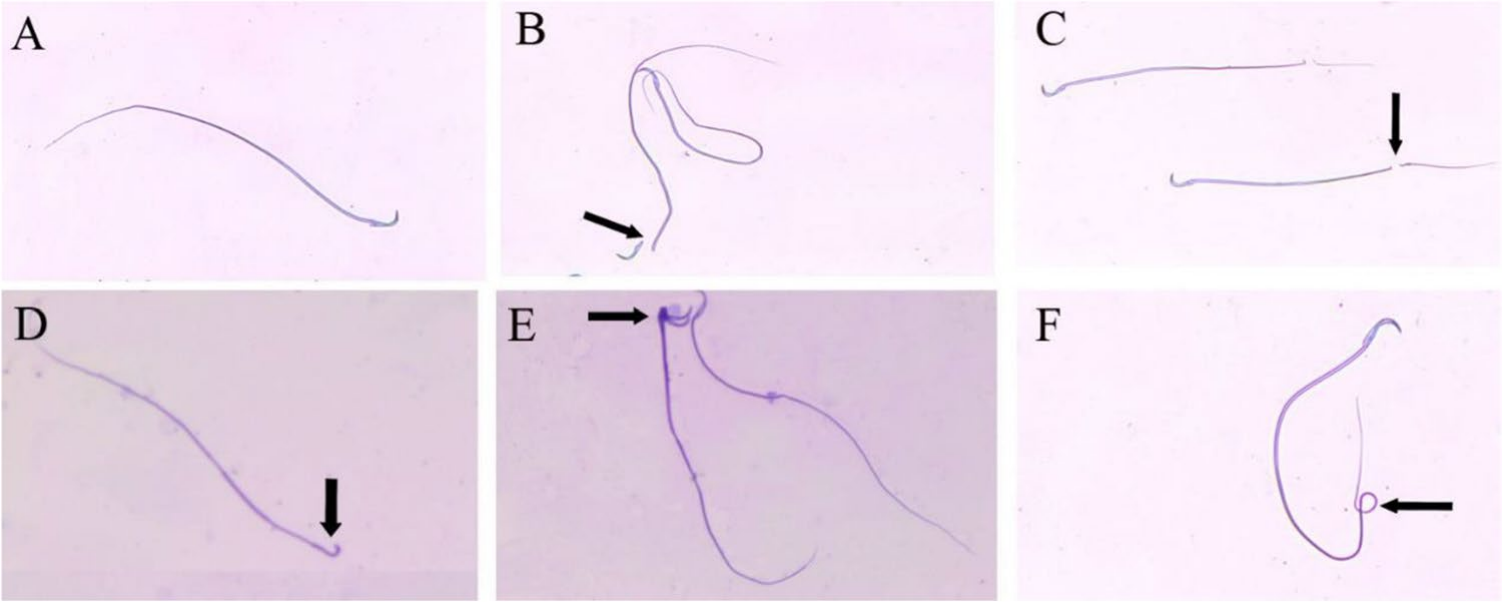
Photos of methyl violet stained semen film of control and SFX-treated rats showing epididymal sperms morphology. (**A**) Normal sperm, (**B**) detached head, (**C**) broken tail, (**D**) microcephalic head, (**E**) double head with a single tail, and (**F**) coiled tail (× 40)

**Fig. 3:**
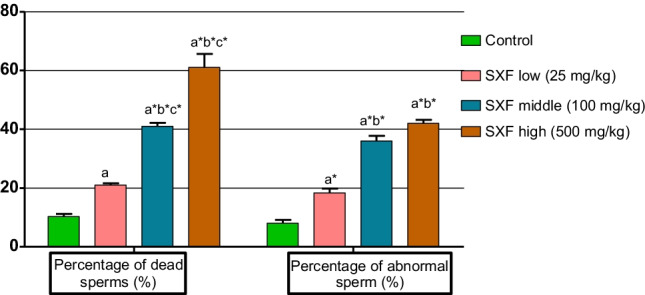
The effect of SFX on sperm viability and morphology in male rats exposed to 25, 100, and 500 mg/kgb.w. of SFX for 4 weeks. * indicates highly significance at *p* ≤ 0.01; a indicates significance at *p* ≤ 0.05 in comparison with the control group; b Indicates significance at *p* ≤ 0.05 in comparison with 25 mg/kg dose group; c indicates significance at *p* ≤ 0.05 between 100 and 500 mg/kg dose groups

**Fig. 4:**
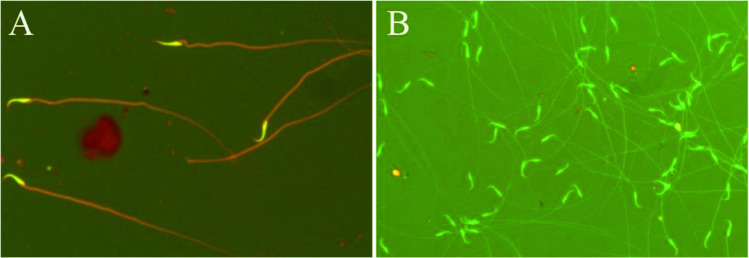
**A** Acridine orange-stained semen film of SFX-exposed rats showed damaged sperm DNA (yellow head). **B** Semen film of control rats showed normal intact sperm DNA (appeared green head) (× 40)

The percentage of dead epididymal sperm showed a significant increase at 25 (*P* < 0.05), 100 and 500 mg/kg doses (*P* < 0.01) groups as compared with the control group. Also, 100 and 500 mg/kg SFX dose groups revealed a significant (*P* < 0.01) increase as compared with 25 mg/kg dose group. However, there is a significant (*P* < 0.01) increase in 500 mg/kg SFX dose group in comparison with 100 mg/kg dose group (Figs. 1 and 3). The percentage of abnormal sperms significantly (*P* < 0.01) increased in all SFX-exposed groups as compared with the control group, while 100 and 500 mg/kg dose groups showed a significant (*P* < 0.01) increase in comparison with the 25 mg/kg dose group (Figs. 2 and 3). The percentage of DNA damage in sperms of rats in the 500 mg/kg dose group indicated a significant (*P* < 0.01) increase in comparison with control, 25, and 100 mg/kg doses groups (Table 4) (Figs. 3 and 4).

**Table 4:** The percentage of intact and damaged sperms of rats exposed to 25, 100, and 500 mg/kg/b.w.of SFX for 4 weeks

Groups	Intact sperm (%)	Damaged sperm (%)
Control	100 ± 0.000	0.000 ± 0.000
25 mg/kg SFX	100 ± 0.000	0.000 ± 0.000
100 mg/kg SFX	98.666 ± 0.333c*	1.333 ± 0.333c*
500 mg/kg SFX	90.000 ± 1.732a*b*c*	10.000 ± 1.732a*b*c*

Values were expressed as means ± SE (*n* = 10). * indicates highly significant at *p* ≤ 0.01; a indicates significance at *p* ≤ 0.05 in comparison with the control group; b indicates significance at *p* ≤ 0.05 in comparison with 25 mg/kg dose group; c indicates significance at *p* ≤ 0.05 between 100 and 500 mg/kg dose groups

### Histopathological examination

**Fig. 5:**
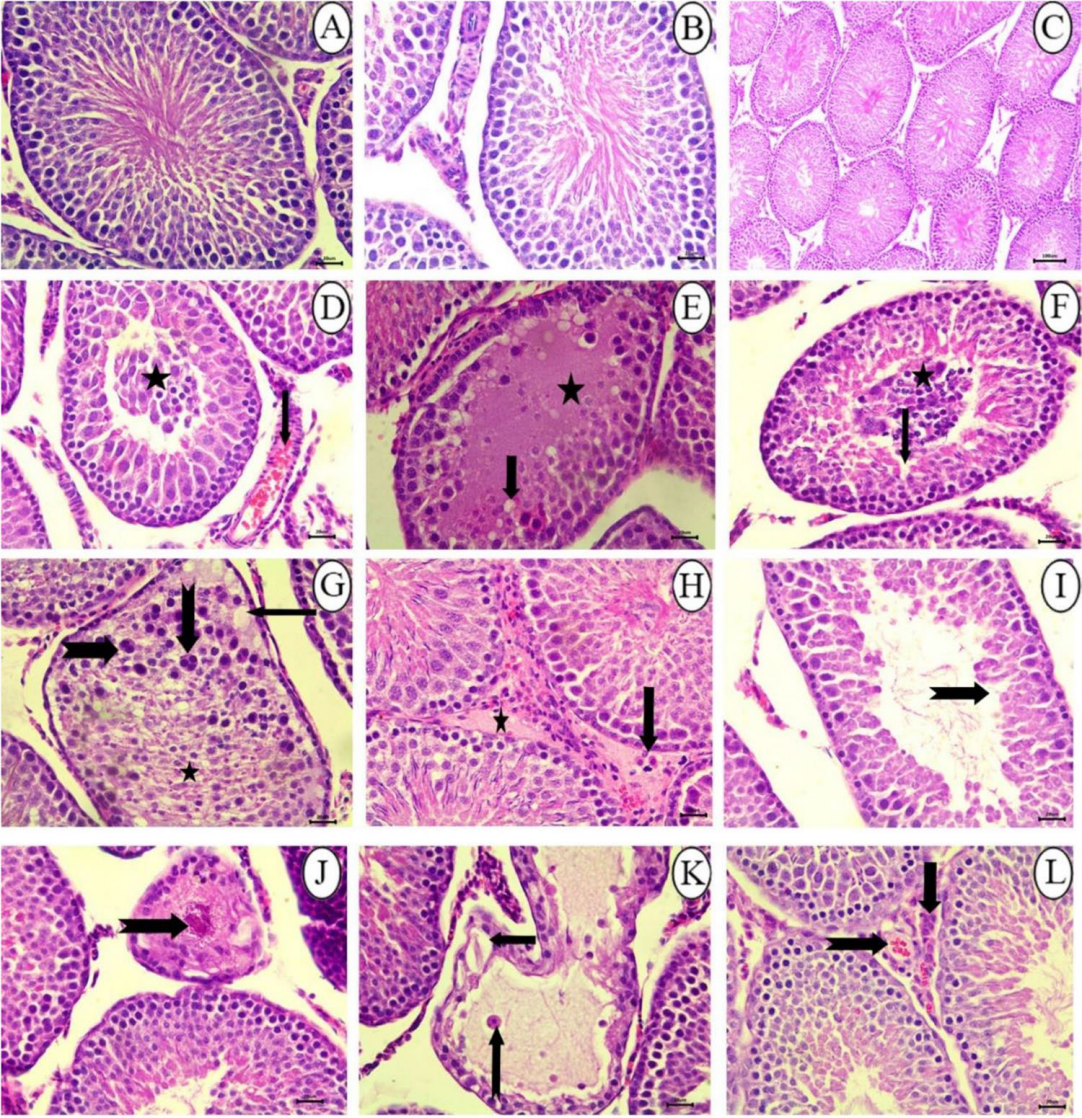
Histopathological examination of testes in (**A** and **B**) control group showing seminiferous tubules lined by normal germinal epithelium, bar = 20 μm; (**C**) control group showing seminiferous tubules, bar = 100 μm; (**D**) 25 mg/kg SFX-exposed group showing a collection of tissue debris inside the lumen of seminiferous tubules (star) and hyperemia (arrow), bar = 20 μm; (**E**) 25 mg /kg SFX-exposed group showing necrosis of germinal epithelium (arrow) and lumen filled with oedematous fluid (star), bar = 20 μm; (**F**) 100 mg/kg SFX-exposed group showing (spermatid giant cells) (star), degeneration of germinal epithelium (arrow), bar = 20 μm; (**G**) 100 mg/kg SFX-exposed group showing hypereosinophilic disorganized cells (star), vacuolation (arrow), and formation of (spermatid giant cells) (notched arrow), bar = 20; (**H**) 100 mg/kg SFX-exposed group showing interstitial oedema (star) with mild hyperplasia of (Leydig cells) (arrow), bar = 20 μm; (**I**) 100 mg/kg SFX-induced group showing necrosis of germinal epithelium (notched arrows), bar = 20 μm; (**J**) 500 mg/kg SFX-induced group showing coagulative necrosis in germinal epithelium (notched arrow), bar = 20 μm; (**K**) 500 mg/kg SFX-exposed group showing detachment of germinal epithelium (arrow) with the presence of eosinophilic fluid in the lumen of seminiferous tubules (star) and multinucleated giant cells (notched arrow), bar = 20; and (**L**) 500 mg/kg SFX-exposed group showing congestion of interstitial blood vessels (notched arrow) and hyperplasia of (Leydig cells) (arrow), bar = 20 μm, H&E

**Fig. 6:**
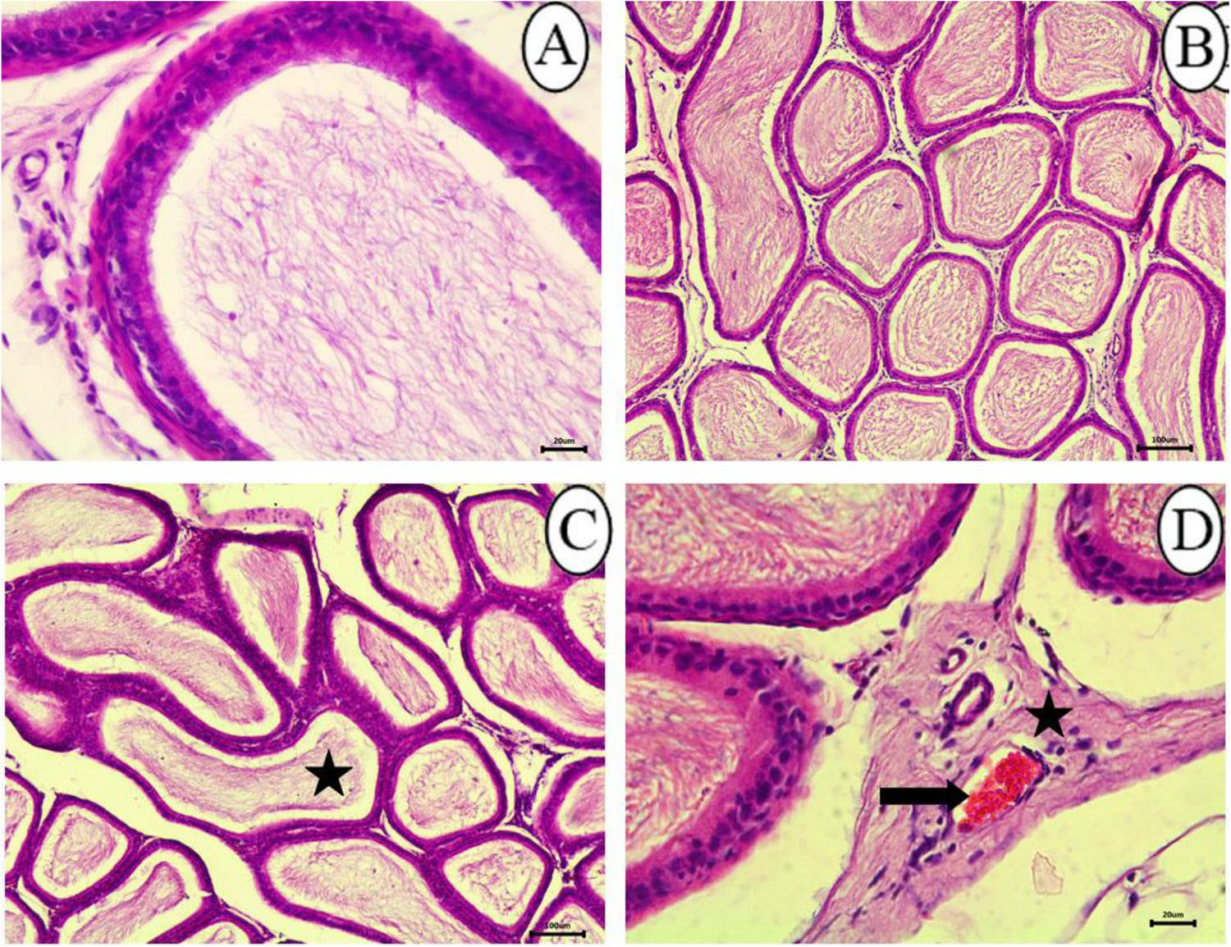
Histopathological examination of epididymis in (**A**) control group showing normal epididymal convoluted tubule lined with pseudostratified columnar epithelium bar = 20 μm; (**B**) control group showing epididymal ducts in, bar = 100 μm; (**C**) 25 mg/kg SFX-exposed group showing decrease in number of sperms inside the epididymal ducts (star), bar = 100 μm; and (**D**) 25 mg/kg SFX-exposed group showing hyperemia (arrow), proliferation of interstitial connective tissue (star), bar = 20 μm, H&E

**Fig. 7:**
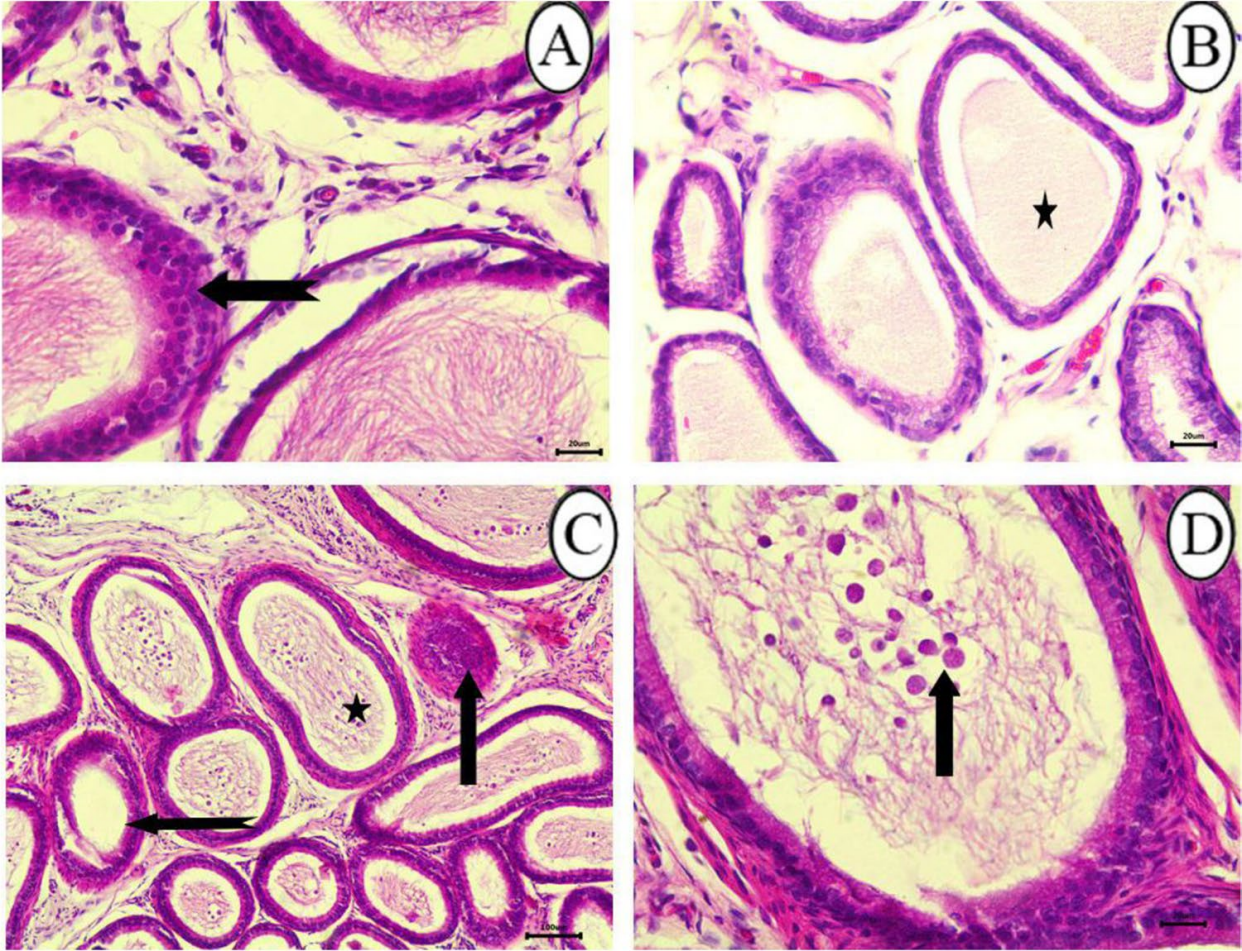
Histopathological examination of epididymis in (**A**) 100 mg/kg SFX-exposed group showing hyperplasia of lining epithelium (notched arrow), bar = 20 μm; (**B**) 100 mg/kg SFX-exposed group showing that the most of epididymal ducts were devoid from spermatozoa (star), bar = 20 μm; (**C**) 500 mg/kg SFX-exposed group showing accumulation of (spermatid giant cells) (star), epididymal duct completely free from spermatozoa (notched arrow), and complete necrosis of epididymal duct (arrow), bar = 100 μm; and (**D**) 500 mg/kg SFX-exposed group showing accumulation of (spermatid giant cells) (notched arrow); bar = 20 μm, H&E

**Fig. 8:**
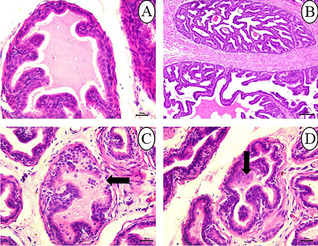
Histopathological examination of seminal vesicles in (**A**) control group showing folded mucosa lined with secretory epithelium, bar = 20 μm; (**B**) the seminal vesicles in control group, bar = 100 μm; (**C**) 25 mg/kg SFX-exposed group showing degeneration and sloughing of lining epithelium (arrow), bar = 20 μm; (**D**) 25 mg/kg SFX-exposed group showing hyperplasia of lining epithelium (arrows), bar = 20 μm, H&E

**Fig. 9:**
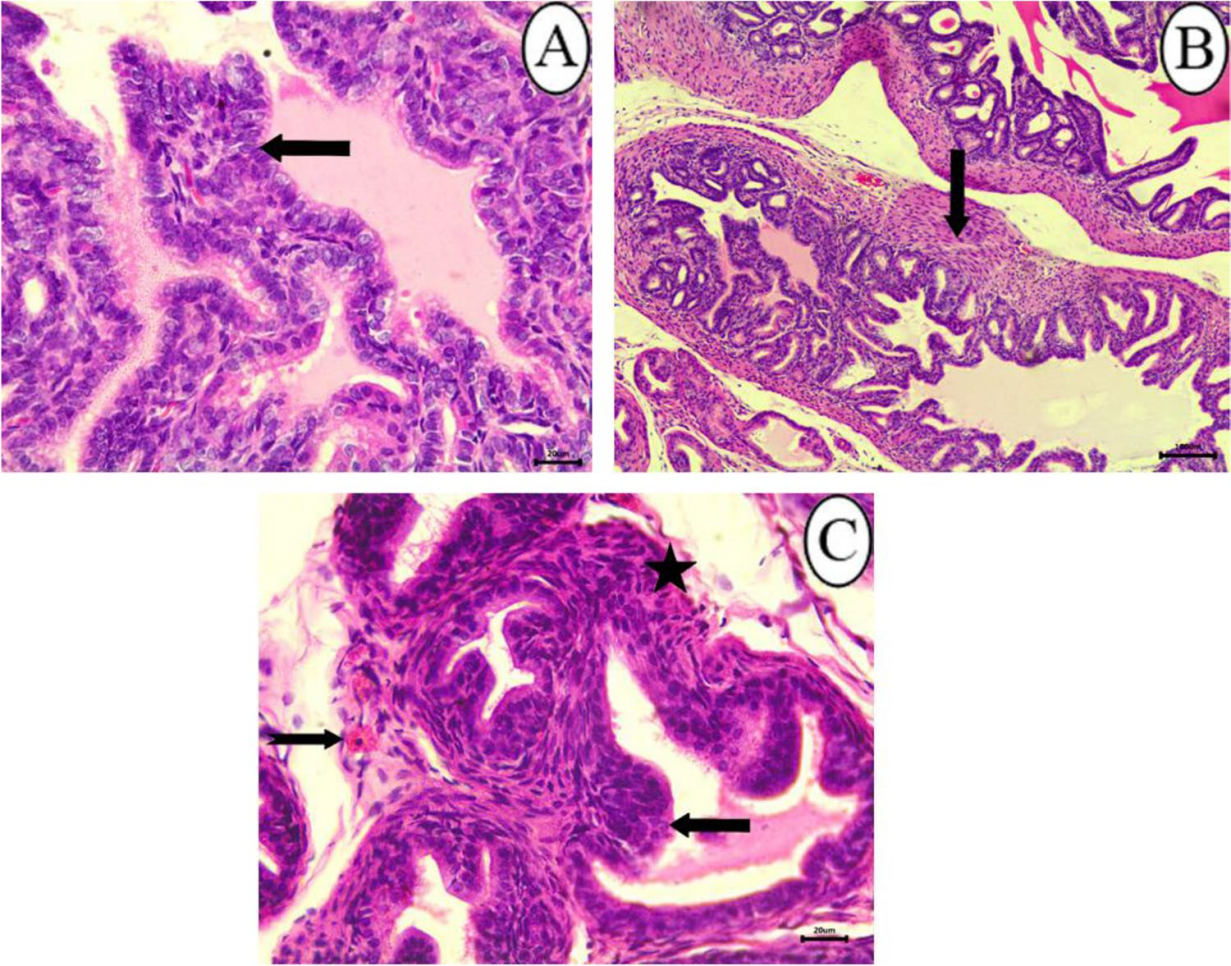
Histopathological examination of seminal vesicles in (**A**) 100 mg/kg SFX-exposed group showing papillary structure extending into the glandular lumen (arrow), bar = 20 μm; (**B**) 100 mg/kg SFX-induced group showing increased prominence of the contracted fibromuscular stroma (arrow), bar = 100 μm; (**C**) 500 mg/kg SFX-exposed group showing hyperplasia of lining epithelium (arrow), connective tissue proliferation in lamina propria (star), and hemorrhage (notched arrow), bar = 20 μm, H&E

Histopathological changes in all SFX-exposed groups are presented in Figs. 5–9 and Table 5. Microscopic examination of testes in the control rats showed normal structure of seminiferous tubules lined by normal germinal epithelium composed of maturing germ cells (spermatogonia, spermatocytes, spermatids, and spermatozoa) and Sertoli cells resting on the basement membrane (Fig. 5A, B, & C). While the histopathological examination of testes in the 25 mg/kg SFX exposed group showed the accumulation of tissue debris with testicular degeneration in the germinal epithelium causing decrease in the number of layers in the lining epithelium in a small number of seminiferous tubules (Fig. 5D). Dilatation of seminiferous tubules was also noticed in other tubules, characterized by a necrosis in the lining germinal epithelium with an increased luminal diameter of the seminiferous tubules filled with oedematous fluid (Fig. 5E). Exposure to 100 mg/kg SFX induced obvious damage in the testes of the rats. This damage characterized by testicular degeneration appeared in most seminiferous tubules with the appearance of multinucleated giant cells in the tubular lumen associated with a decrease in the number of layers of germinal epithelium lining of seminiferous tubules (Fig. 5F). Another pattern of the testicular deformity was necrosis in most layers of the germinal epithelium with hypereosinophilic, vacuolized, disorganized cells showing pyknosis of nucleoli accompanied with the formation of (spermatid giant cells) (Fig. 5G). Additionally, interstitial oedema with mild hyperplasia of (leydig cells) was seen in 75% of rats, in a focal manner (Fig. 5H). Dilatation of seminiferous tubules with necrosis of the germinal epithelium was seen in sporadic seminiferous tubules (Fig. 5I). Rats exposed to 500 mg/kg SFX showed evident histopathological alterations. Coagulative necrosis in germinal epithelium of the seminiferous tubules with absence of mature sperms in the lumen was noticed in many tubules (Fig. 5J). Detachment of germinal epithelium with dilatation of seminiferous tubules was filled with eosinophilic fluid with the appearance of multinucleated giant cells (Fig. 5K). Congestion of interstitial blood vessels with hyperplasia of Leydig cells also was seen in testes of rats in this group (Fig. 5L).

**Table 5:** Incidence of histopathological lesions in testis, epididymis, and seminal vesicle of the experimental groups

Lesions	Control	25 mg/kg SFX	100 mg/kg SFX	500 mg/kg SFX
Testis:				
Congestion	- (100%)	+ + (50%)	+ + + (75%)	+ + + + (100%)
Interstitial edema	- (100%)	+ + (75%)	+ + + (75%)	+ + + + (100%)
Leydig cells hyperplasia	- (100%)	- (100%)	+ + (25%)	+ + (50%)
Degeneration and necrosis of seminiferous tubules	- (100%)	+ + + (25%)	+ + + + (50%)	+ + + + (75%)
Intratubular giant cell formation	- (100%)	+ + + (25%)	+ + + + (50%)	+ + + + (75%)
Epididymis:				
Congestion	- (100%)	+ + (50%)	+ + + (75%)	+ + (100%)
Interstitial C.T. proliferation	- (100%)	+ + (25%)	+ + + (25%)	+ + + (50%)
Hyperplasia of lining cells	- (100%)	- (25%)	+ + (50%)	+ + (50%)
Intratubular giant cell formation	- (100%)	- (100%)	- (100%)	+ + (25%)
Disappearance of sperms in the lumen of epididymal ducts	- (100%)	+ (25%)	+ + (50%)	+ + + (25%)
Seminal vesicle:				
Degeneration and sloughing of the lining epithelium	- (100%)	+ (25%)	+ (25%)	+ (25%)
Hyperplasia of lining cells	- (100%)	+ (25%)	+ (25%)	+ + (50%)
Atrophy of seminal vesicles	- (100%)	- (100%)	+ (25%)	+ + (50%)
Hemorrhage	- (100%)	- (100%)	+ (25%)	+ (25%)

- no lesions; + slight lesions; +  + moderate lesions; +  +  + severe lesions; +  +  +  + very severe lesions. Percentages represent the no. of affected rats in each group

Histological examination of the epididymides in control rats showed an epididymal convoluted tubule lined with pseudostratified columnar epithelium (Fig. 6A&B). The changes of epididymis in the 25 mg/kg SFX-intoxicated group included decrease in the number of sperms inside the lumen of epididymal ducts **(**Fig. 6C**)**; proliferation of interstitial connective tissue with congestion of intertubular blood vessels were observed in the epididymis of this group **(**Fig. 6D**)**. Microscopic examination of epididymal sections in the 100 mg/kg SFX-intoxicated group revealed that all examined rats exhibited obvious changes. These changes were expressed by hyperplasia of lining epithelium **(**Fig. 7A**)**. Exposure to 500 mg/kg SFX induced complete decrease of sperms in epididymal lumen **(**Fig. 7B**)**. Complete absence of sperms in some epididymal ducts while others complete necrosed accompanied with an accumulation of spermatid giant cells inside most of the epididymal ducts were the most detectable lesions in this group **(**Fig. 7C, D**)**.

The seminal vesicles of control rats were normal in architecture. The walls of seminal vesicles were folded mucosa lined with secretory epithelium **(**Fig. 8A&B**)**. Degeneration and sloughing of the lining epithelium of seminal vesicles were the most noticeable lesions in the 25 mg/kg SFX-intoxicated group **(**Fig. 8C**)**. While in other seminal vesicles, there was focal hyperplasia of lining epithelium **(**Fig. 8D**)**. Atrophy of seminal vesicles was a distinctive feature, predominated in 25% of examined rats in the 100 mg/kg SFX-intoxicated group. Atrophy of seminal vesicles tends to appear microscopically as a decrease in the size of glandular components with increased prominence of the contracted fibromuscular stroma accompanied by hyperplasia of glandular epithelium forming small papillary folds in the glandular lumen **(**Fig. 9A, B**)**. In the 500 mg/kg SFX-exposed group, hyperplasia of lining epithelium associated with interstitial connective tissue proliferation in lamina propria with focal areas of hemorrhage were observed in the 50% of examined rats **(**Fig. 9C**)**.

## Discussion

Previous reports mentioned that the use of neonicotinoids has several toxicity outcomes such as neurotoxicity, hepatotoxicity immunotoxicity, and nephrotoxicity in both vertebrates and invertebrates **(**Han et al. 2021). Despite the current evidence that demonstrated different hazards for SFX application, its potential impact on the male reproductive system remains unclear.

In the current study, exposure of rats to 500 mg/kg SFX for 4 weeks lead to a significant increase in FSH level in comparison with the control and 25 mg/kg dose groups, while LH level revealed a significant increase at 100 mg/kg group and 500 mg/kg SFX dose groups as compared with the control group. This is in agreement with Saber et al. (2021) who showed that exposure of male rats to imidacloprid at a dose of 22.5 mg/kg every day for 56 days caused an increase in the levels of LH and FSH compared to the control group. Similarly, another study conducted on exposure of the rats to acetamiprid with low (12.5 mg/kg), medium (25 mg/kg), or high dose (35 mg/kg) for 90 days caused an increment of serum levels of FSH and LH in low- and medium-dose groups (Arıcan et al. 2020).

A similar study investigated the effects of the most common insecticides used in Nigeria on the reproductive hormones of male rats and found that the insecticides caused an increase in FSH levels compared to control rats (Airaodion et al. 2019). Regulation of spermatogenesis in the testes occurs at the hypothalamic-pituitary–testicular axis. Gonadotropin-releasing hormone (GnRH) stimulates gonadotropin cells to secrete LH and FSH. GnRH release is regulated by testosterone through a negative feedback loop. Luteinizing hormone stimulates Leydig cells to produce testosterone and FSH stimulates Sertoli cells to produce androgen-binding protein (ABP). Androgen-binding protein binds to testosterone to enhance meiosis of the spermatocytes (Zhou et al. 2019). In the current study, the high-serum measure of gonadotrophins with exposure to SFX prompted to suppression of feed-back inhibition of the anterior pituitary. The suppression of feedback inhibition may lead to an increase the LH and FSH secretion (Zhou et al. 2019). These results indicated that SFX has a direct effect on the pituitary, which leads to an increase in circulating LH levels. The results obtained for serum FSH and LH levels after exposure to SFX with different doses agree with the findings of Darbre (2018) who reported that air pollution has a serious effect on hormones, because many environmental pollutant chemicals can interfere with the function of the endocrine system, which are called endocrine disrupting chemicals. However, the exact mechanism of how these chemicals affect hormones is still unclear. In the present study, the level of MDA in rats significantly increased in all SFX-exposed groups as compared with the control group. Also, GPx activity indicated a significant increase in 100 and 500 mg/kg dose groups as compared with the control group and 25 mg/kg dose group. This coincides with Fathy and Abdelkader (2021b) who noticed a significant dose-dependent increment of MDA level in the spleen of SFX-treated groups as compared with the control group. Sahinoz et al. (2019) has also previously shown that chlorpyrifos insecticide exposure increased the production of free radicals and induced oxidative stress. A previous study evaluated the biochemical effect of gestational exposure to four pesticides on female Wistar rats and their offspring at adult age and indicated that MDA levels were significantly higher in pesticide-exposed rats compared to the control group (Ndonwi et al. 2019). Malondialdehyde (MDA) is a major oxidation product of polyunsaturated fatty acids and increased MDA content is a critical indicator of lipid peroxidation. In our study, the MDA level increased in SFX-treated rats. It may be proposed that increment of the MDA level can be an indicator of SXF lipoperoxidative injury. Also, another study that investigated the acute toxicity of pesticides as profenofos, cyhalothrin, and azadirachtin showed that GPx depicted an initial increment followed by a subsequent reduction (Chatterjee et al. 2021). Similarly, Piner Benli and Çelik (2021a) described that GPx activity and MDA level increased in sulfoxaflor-treated group compared to the control group. In our study, activity of GPx increased in SFX-exposed rats. The increase in GPx activity may be due to an increase in the production of reactive oxygen species in SFX toxicity.

Hormonal regulation of the male reproductive system can be altered by the imbalance between the production of reactive oxygen species and the antioxidant defense mechanisms. An increase in the generation of reactive oxygen species may damage reproductive tissues or intervene with the hypothalamic-pituitary gonadal axis and its connections with other endocrine axis and badly affect male reproductive function, thus inducing infertility in males (Darbandi et al. 2018). From their study, the generation of reactive oxygen species caused activation of the hypothalamic-pituitary axis and released cortisol (in humans) in response to stress. These stress hormones adversely affect LH secretion from the anterior pituitary. Therefore, the adverse impact of SFX on male sex hormones in this study could be attributed to the production of free radicals and the induction of oxidative stress.

In our study, the percentage of dead and abnormal epididymal sperm showed a significant increase in all SFX-exposed groups as compared with the control group. The percentage of DNA damage in sperms of rats after exposure to SFX at 500 mg/kg for 4 weeks also indicated a significant increase in comparison with the control, 25, and 100 mg/kg dose groups**.** This is in tandem with another study which also observed that rats had lower sperm counts after 45 days of acetamiprid and these rats revealed a significant decrease in sperm motility and increased rates of abnormal morphology (Mosbah et al*.* 2018). Exposure to neonicotinoid insecticides as acetamiprid at 26.67, 40, and 80 mg/kg body weight for 90 days leads to a significant reduction in sperm motility and count of epididymal sperm and a significant increment in the rate of abnormal sperms when compared to the control group (Kenfack et al. 2018). However, Quinalphos exposure at 1.0 mg/kg resulted in a significant reduction in motility and increment in sperm head defects and DNA damage (Kumari et al. 2021). Pesticides including SFX induce oxidative stress led to imbalance of antioxidant defense system (Bhardwaj et al. 2018). In this study, the increased level of MDA in serum indicates that SFX caused oxidative stress. Elevated oxidative stress induced sever changes in the epididymis and maturing spermatozoa that result in the impairment of sperm quality (Wu et al. 2020). Moreover, oxidative damages in sperms are associated with immobility, reduced fertilization rate, DNA damage, and high frequency of sperm apoptosis (Mehraban et al. 2019). In the current study, an increment in abnormal sperm morphology and DNA damage observed after 4 weeks after SXF administration revealed that SFX has more adverse effects on male reproductive tissue. Furthermore, another way SFX affects the function of the male reproductive system is to damage DNA (Said et al. 2021), since the morphology of sperm is organized by various autosomal and Y-specific genes (Bansal et al. 2017).

Histopathological investigation of testes in 25 mg /kg SFX-intoxicated group showed testicular degeneration and a decrease in the number of layers that appeared in a small number of seminiferous tubules with dilatation of seminiferous tubules filled with eosinophilic fluid. The testicular damage was increased in the rats intoxicated with 100 mg /kg SFX which was characterized by testicular degeneration that appeared in most of the seminiferous tubules, dilatation of seminiferous tubules accompanied with hypereosinophilic disorganized vacuolated cells. Additionally, interstitial edema with mild hyperplasia of Leydig cells was also seen. The same alterations were seen in animals treated with 500 mg /kg SFX but became more obvious described as severe coagulative necrosis in the cells of lining layers in the seminiferous tubules. Similar results described by Zakzook et al. (2020) who reported on the effect of dimethoate in testes characterized by degeneration, necrosis, and loss of spermatogenic cell layer associated with Sertoli cell vacuolation and severe interstitial edema. Hyperplasia of Leydig cells in both 100 mg/kg SFX-exposed group and 500 mg/kg SFX-exposed group explain the increase in the testosterone hormone in these groups. Concerning histopathological observations of epididymis was expressed as the proliferation of interstitial connective tissue infiltrated with inflammatory cells with congestion of intertubular blood vessels in all intoxicated groups. The epididymal damage became more evident in the epididymis of 500 mg/kg SFX-exposed rats which was characterized by the accumulation of spermatid giant cells with a decrease in sperm concentration in the epididymal lumen. These results agree with the results appeared in the study used mixture of the pesticides (cypermethrin, mancozeb, and metalaxyl); pesticides revealed decrease epididymis mass by reducing sperm concentration and the degenerative change in epididymal tissues (Bouabdallah et al. 2022) According to Ali and Ibrahim (2018), the harmful effects of some pesticides on reproductive system of may be due to its electrophilic attack on their cells. Degeneration of lining epithelium of seminal vesicles was observed in the 25 mg/kg SFX-intoxicated group. Seminal vesicles completely atrophied in the 100 mg/kg SFX-intoxicated group in some cases. Damage to accessory organs could be attributed due to a significant increase in MDA and GPx levels, while focal areas of hyperplasia of lining epithelium in other seminal vesicles may be related to an increase of testosterone level (Creasy et al. 2012).

## Conclusion

Sulfoxaflor induced biochemical changes in FSH, LH, MDA, and GPx in serum of rats. Also, sulfoxaflor increased dead and abnormal sperm percentage and caused DNA damage in sperms of rats in comparison with the control group. Histopathological examination of testes showed testicular degeneration with severe coagulative necrosis while epididymis revealed the proliferation of interstitial connective tissue infiltrated with inflammatory cells with congestion of intertubular blood vessels as well as degeneration of lining epithelium of seminal vesicles.

## Data Availability

Applicable.
